# PREVALENCE OF HEPATIC STEATOSIS AMONG CHILDREN AND ADOLESCENTS WITH CYSTIC FIBROSIS AND ITS ASSOCIATION WITH NUTRITIONAL STATUS

**DOI:** 10.1590/1984-0462/;2019;37;4;00007

**Published:** 2019-06-19

**Authors:** Amanda Oliva Gobato, Ana Carolina Junqueira Vasques, Antonio Fernando Ribeiro, Roberto Massao Yamada, Gabriel Hessel

**Affiliations:** a Universidade Estadual de Campinas, Campinas, SP, Brazil.

**Keywords:** Cystic fibrosis, Fatty liver, Malnutrition, Nutritional status, Child, Adolescent, Fibrose cística, Esteatose hepática, Desnutrição, Estado nutricional, Criança, Adolescente

## Abstract

**Objective:**

To determine the prevalence of hepatic steatosis (HS) in children and adolescents with cystic fibrosis (CF) and associate it with nutritional status.

**Methods:**

Cross-sectional study with children and adolescents with CF diagnosis. Weight and height were used to calculate the body mass index (BMI) and subsequent classification of the nutritional status. The midarm circumference (MAC), triceps skinfold thickness (TSF) and midarm muscle circumference (MAMC) were used to evaluate body composition. Abdominal ultrasonography was performed for diagnosis of HS. The statistical tests used were Student’s t test, Mann-Whitney test and chi-square test with significance level of 5%.

**Results:**

50 patients with CF were evaluated, 18 (36%) were diagnosed with HS (Group A) and 32 (64%) without HS (Group B). The mean age of Group A was 13,2±4,9 years old and Group B 11,7±4,9; for BMI, the value for Group A was 18,0±4,1 and Group B was 15,7±3,8; the TSF of Group A was 8,4±3,5 mm and Group B was 7,0±2,5 mm. For these variables, there was no significant difference between the groups. The mean of MAC and MAMC differed significantly between the groups, being higher in the HS group, with p values of 0,047 and 0,043.

**Conclusions:**

The frequency of HS in patients with CF is high and it is not related to malnutrition, according to the parameters of BMI, TSF and MAMC. The values of MAC and MAMC indicated a greater reserve of muscle mass in patients with HS.

## RESUMO

**Objetivo:**

Determinar a prevalência de esteatose hepática (EH) em crianças e adolescentes com fibrose cística (FC) e associá-la com o estado nutricional.

**Métodos:**

Estudo transversal com crianças e adolescentes com diagnóstico de FC. Foram aferidos o peso e a altura para o cálculo do índice de massa corpórea (IMC) e classificação do estado nutricional. A circunferência do braço (CB), a dobra cutânea tricipital (DCT) e a circunferência muscular do braço (CMB) foram empregadas para avaliação da composição corporal. A ultrassonografia abdominal foi realizada para o diagnóstico de EH. Os testes estatísticos empregados foram o teste *t* de Student, o teste de Mann-Whitney e o teste do qui-quadrado, com nível de significância de 5%.

**Resultados:**

Dos 50 pacientes avaliados, 18 (36%) apresentaram EH (Grupo A) e 32 (64%) não (Grupo B). Para as médias de idade (Grupo A: 13,3±5,0 anos; e Grupo B: 11,7±5,0 anos), IMC (Grupo A: 18,0±4,1; e Grupo B: 15,7±3,8) e DCT (Grupo A: 8,4±3,5 mm; e Grupo B: 7,0±2,5 mm), não houve diferença significativa entre os grupos. A média da CB e da CMB diferiram significativamente entre os grupos, sendo mais elevada no grupo com EH, com valores p respectivos de 0,047 e 0,043.

**Conclusões:**

É alta a frequência de EH em pacientes com FC e ela não está relacionada com a desnutrição, segundo os parâmetros de IMC, DCT e CMB. Os valores de CB e CMB indicaram maior reserva de massa muscular nos pacientes com EH.

## INTRODUCTION

Recently, attention has been given to hepatic involvement in cystic fibrosis (CF), as this is one of the main causes of death, respiratory failure and complications related to lung transplantation.^1^ CF is the most common potentially fatal genetic disease in the white race, with incidence of approximately one per three thousand live births.^2^ It is a multisystemic disease affecting the sweat glands, pancreas, lungs, livers, intestines and Wolff ducts.^3^ The diagnosis of CF is made by liver test and is confirmed when chloride concentration is higher than 60 mEq/L in two exams conducted on different days.^4^

There are several reasons that hamper understanding the actual prevalence of liver disease in CF: there is no unanimously accepted definition of criteria for diagnosis of liver disease in CF, most patients are asymptomatic, and highly sensitive and non-invasive tests are scarce. The term hepatic disease in CF is non-specific and has been used in several studies to describe a broad spectrum of hepatobiliary diseases such as: neonatal cholestasis, elevated liver enzymes, imaging abnormalities including liver parenchymal heterogeneity upon ultrasonography, portal hypertension, liver failure and histological abnormalities such as fibrosis, cirrhosis, and hepatic steatosis (HS).^5^ Cirrhosis and portal hypertension occur in 5 to 8% of patients and, in most cases, onset in the first decade of life.^6^

HS is reported in a case series with frequency of 20-60%.^7^ Although the etiopathogenesis in most patients is unknown, it has been associated with specific nutritional deficiencies, altered phospholipid metabolism,^8^ and malnutrition.^9^ Essential fatty acid deficiency has been described in patients with CF and pancreatic insufficiency,^10^ while experimental studies with rats have linked this deficiency to HS.^11^ According to the consensus body of hepatobiliary diseases related to CF, when patients without malnutrition present with steatosis, it is important to investigate the possibility of diabetes mellitus.^12^ On the other hand, the pathophysiology of malnutrition in HS is very little understood. Van Zutphen et al.^13^ induced severe malnutrition in rats and showed that the main mechanisms leading to HS are loss of peroxisome and mitochondrial dysfunction.

In this context, this study aimed to describe the prevalence of HS found by abdominal ultrasonography in children and adolescents with CF and associate it with nutritional status.

## METHOD

This was a cross-sectional study carried out with 50 children and adolescents aged between 2 and 19 years of age, with diagnosis of CF established by two sodium and chloride dosages in sweat above 60 mEq/L.^4^

Inclusion criteria were: patients assisted at the Cystic Fibrosis Outpatient Clinic of Clinical Hospital of the School of Medical Sciences, *Universidade Estadual de Campinas* (UNICAMP), Campinas, SP, Brazil, in 2016, and whose caregivers signed the free informed consent form. Exclusion criteria were: patients on hepatotoxic drugs with elevated aminotransferases, severe dyslipidemia, or other liver diseases that may occur with HS (viral hepatitis B and C infections, alpha-1 antitrypsin deficiency, and Wilson’s disease).

Weight, height, midarm circumference (MAC) and triceps skinfold thickness (TSF) were measured by the anthropometric techniques recommended by Lohman. A Lange skinfold measure was used to measuring TSF, and a Sanny tape was used to measure MAC. The body mass index (BMI)/age was calculated using the Quetelet index (BMI=weight/height^2^), and BMI was classified according to the World Health Organization (WHO) growth curves.^14^ Patients with percentile <3 were classified as malnourished, patients with percentile ≥3 and <85 were considered eutrophic, and overweight was linked to percentile ≥85.

With the values obtained from TSF and MAC, the midarm muscle circumference (MAMC) was calculated, according to Equation 1:

MAMC=AC-(0.314×TSF) (1)

The percentage of adequacy was measured according to Equation 2:

MAMC or TSF/percentiles 50×100 (2)

Patients with values ≤90% were classified as malnourished, while eutrophic patients were those whose percentile resulted >90. Patients with presenting values >110% were considered as having excessive body fat only for TSF.^15^

**Figure 1:**
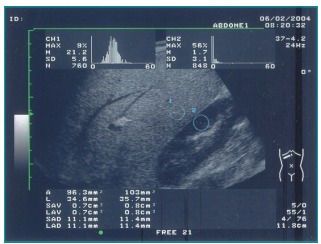
Ultrasonography of the liver in a patient with hepatic steatosis. The hepatorenal contrast is moderate, and the difference in histogram of the right hepatic lobe/renal cortex is 19.5.

Abdominal ultrasound is the most commonly used imaging method to identify HS because it is relatively low-cost, non-invasive, easy to apply and available in most services. The sensitivity of the method was 89%, and specificity was 93%.^16^ Ultrasound was performed using a Toshiba Power Vision 6000 device at 3.75 MHz and 5 MHz linear array transducers by two examiners experienced in pediatric abdominal ultrasonography. The patient remained in supine position for evaluation of the liver after a 12-hour fast. Diagnosis of HS was considered in case of moderate or severe hepatorenal contrast and/or difference of ≥7 in histogram of the right lobe/right kidney cortex ratio,^17^ as shown in Figure 1.

Pancreatic insufficiency was considered present in patients using pancreatic enzymes and/or presenting with steatorrhea. Diabetes mellitus was considered according to the classification by the Brazilian Guidelines for the Diagnosis and Treatment of Cystic Fibrosis.^18^

The method for selection of participants was convenience sampling; the 85 patients cared for in 2016 were invited to participate and 50 of them met the inclusion and exclusion criteria. Patients were divided into two groups according to ultrasound results:

- Group A: patients with CF and HS.
- Group B: patients with CF and without HS.

Data were analyzed in the IBM Statistical Package for the Social Sciences (SPSS) software version 20.0. The Kolmogorov-Smirnov test was applied to evaluate the distribution of quantitative variables in Gaussian curve. The descriptive analysis of continuous variables included calculation of means and respective standard deviations for variables with normal distribution, and calculation of medians and 25th and 75th percentiles for variables that did not adhere to the normality test. Categorical variables were expressed in percentage values. Student’s t-test was used to compare the variables with normal distribution according to the presence of HS; for variables without normal distribution, the Mann-Whitney test was applied. The chi-square test was used to investigate the association between HS and categorized anthropometric indicators. As some variables were allocated in more than two categories, the verification of local association between categories was analyzed by calculating adjusted residuals. Adjusted residual values greater than 1.96 would indicate a statistically significant association between both categories.

This study was approved by the Research Ethics Committee of the School of Medical Sciences of UNICAMP, protocol 494,781.

## RESULTS

Fifty patients from the Cystic Fibrosis Outpatient Clinic of UNICAMP Clinical Hospital were evaluated, being 23 (46%) females and 27 (54%) males aged between 2 and 19 years of age (12.2±4.9); there were four children and 14 adolescents in Group A, 13 children and 19 adolescents in Group B, considering the classification for children aged <10 years.

**Figure 2:**
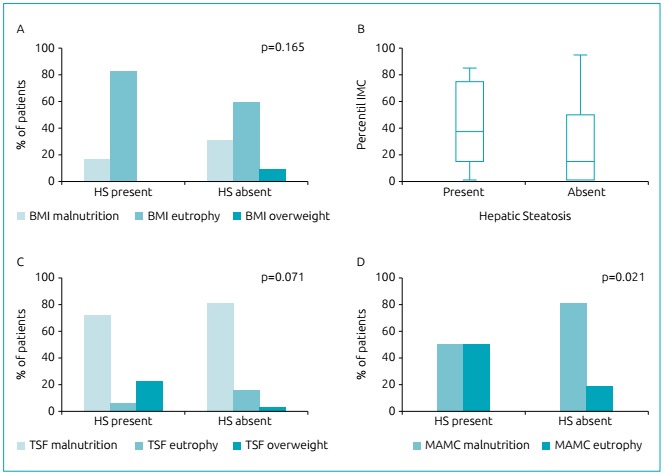
(A) Classification of nutritional status by body mass index according to presence/absence of hepatic steatosis. (B) Distribution of percentiles of Body Mass Index in box plot for patients with and without hepatic steatosis. (C) and (D) Association of triceps skinfold and midarm circumference according to presence/absence of hepatic steatosis.

Eighteen patients (36%) were diagnosed with HS by abdominal ultrasonography (Group A) and 32 (64%) were diagnosed as not having HS (Group B). When evaluating the association between HS and nutritional status, the variables BMI and TSF did not differ significantly between groups, meaning no association between HS and malnutrition. MAC and MAMC were significantly different between groups, with the highest difference in the HS group, indicating greater muscle mass (Table 1 and Figure 2).

**Table 1:** Nutritional status, pancreatic insufficiency and presence of diabetes mellitus in fibrocystic patients with and without hepatic steatosis.

		Hepatic steatosis		p-value	Total
		Present (n=18)	Absent (n=32)		
Age (years) mean*** and SD		13.2±4.9	11.7±4.9	0.291	12.2±4.9
Gender**	Female (%)	8 (44.4)	15 (46.9)	0.869	23 (46)
	Male (%)	10 (55.6)	17 (53.1)		27 (54)
BMI*** (mean and SD)		18.0±4.1	15.7±3.8	0.058	16.6±4.0
BMI classification**	Malnutrition (%)	3 (16.7)	10 (31.2)	0.165	13 (26)
	Eutrophy (%)	15 (83.3)	19 (59.4)		34 (68)
	Overweight (%)	0	3 (9.4)		3 (6)
MAC*** (cm) mean and SD		20.3±6.8	16.5±4.5	0.047*	17.9±5.7
TSF*** (mm) mean and SD		8.4±3.5	7.0±2.5	0.134	7.5±2.9
TSF classification**	Malnutrition (%)	13 (72.2)	26 (81.2)	0.071	39 (78)
	Eutrophy (%)	1 (5.6)	5 (15.6)		6 (12)
	Overweight (%)	4 (22.2)	1 (3.1)		5 (10)
MAMC*** (mm) mean and SD		177.7±61.1	143.3±41.1	0.043*	155.7±51.4
MAMC classification**	Malnutrition (%)	9 (50)	26 (81.2)	0.021*	35 (70)
	Eutrophy (%)	9 (50)	6 (18.8)		15 (30)
Pancreatic insufficiency**	Present (%)	18 (100)	30 (93.8)	0.279	48 (96)
	Absent (%)	0 (0)	2 (6.2)		2 (4)
Diabetes mellitus**	Present (%)	3 (16.7)	4 (12.5)	0.684	7 (14)
	Absent (%)	15 (83.3)	28 (87.5)		43 (86)

SD: standard deviation; BMI: body mass index; MAC: midarm circumference; TSF: triceps skinfold; MAMC: midarm muscle circumference; *p<0.05; **chi-square test for categorical variables; ***Student’s t test for continuous variables.

From all patients evaluated, 48 (96%) had pancreatic insufficiency and seven (14%) had diabetes mellitus and no significant association with presence or absence of HS. These data show that the mean BMI of the HS group (18.0±4.1) was higher when compared to the non-HS group (15.7±3.8), but with no significant statistical difference; 15/18 patients (83.3%) with HS were considered eutrophic (Table 1). When adjusted to BMI percentile mean, the HS group remained as higher (percentile 39.05) than the non-HS group (percentile 27,28), but no significant statistical difference was shown (Table 2).

**Table 2:** Characteristics of the sample according to the percentile (p) of body mass index and per presence/absence of hepatic steatosis.

		HS		p-value*	Total
		Present (n=18)	Absent (n=32)		
Percentile of BMI per age	Median (p25-p75)	38 (15-75)	15 (1-50)	0.175	20 (1-50)
	Mean±SD	39.0±32.1	27.2±30.1		31.5±30.7
	Min-max	1-85	1-95		1-95

BMI: body mass index; SD: standard deviation; *Mann-Whitney test.

## DISCUSSION

There is a broad spectrum of hepatic involvement in CF including HS, with malnutrition as one of the causes involved. In the present study, we report a high frequency of HS, but not related to malnutrition. The exact mechanism of liver disease in CF is not well-known. The primary alteration is known to involve a genetic defect of the cystic fibrosis transmembrane conductance regulator (CFTR) of bile epithelial cells, leading to the production of thick biliary secretion, evolving with biliary ductal obstruction and resulting in the development of fibrosis and biliary cirrhosis.^19^ In the hepatobiliary system, CFTR is expressed in intra- and extrahepatic cholangiocytes, including gallbladder, but not in hepatocytes.^5^ On the other hand, the pathophysiology of HS development is associated with metabolic disorders: increased mobilization of fatty acids from adipose tissue, increased liver fatty acid synthesis, increased triglycerides production, and presence of triglycerides in the liver.^20^

The causes of HS secondary to CF have not yet been fully clarified. The pathogenesis may be related to malnutrition, deficiencies of essential fatty acids, carnitine, choline, oxidative stress, and insulin resistance, and not only to a CFTR gene.^21^ In such circumstances, assessing the deficiency of essential fatty acids and carnitine is necessary, considering that deficiency of these nutrients can lead to HS by decreasing fat metabolism. This condition does not appear to progress to cirrhosis, but this statement may change with further research, given that it has already been proven that non-alcoholic steatohepatitis can progress to cirrhosis in adults.

When evaluating the relationship between HS and BMI/age, 16.7% of patients presented malnutrition, but no significant association with the non-HS group, since 31.2% of patients without HS were malnourished. TSF showed adipose tissue depletion in both groups, but without statistical difference, unlike MAC and MAMC, which were shown significantly different between groups, demonstrating that in the presence of HS the patient maintains a better reserve of lean mass and, consequently, higher MAC. Isolated MAC analysis does not allow to affirm that there is an increase in lean mass, but by analyzing low TSF values, it can be concluded that MAC values reflect higher muscle reserves. This result should be confirmed with larger samples and other methods that evaluate muscle mass.

In the presented series, 13/50 patients (26%) presented malnutrition when classified by BMI/age, regardless of HS. When the mean percentile was evaluated, it was below the recommended (percentile 31.52). The Cystic Fibrosis Foundation^1^ has set the goal for nutritional guidelines that children from 2 to 19 years old should have BMI equal to or higher than the 50th percentile.

According to the publication of 2014 by the European Cystic Fibrosis Society,^22^ which obtains epidemiological records of 35,582 CF patients across Europe, almost half of children and adults with CF were classified as eutrophic. Of the total number of patients followed up, 3,981 (11.1%) presented HS. Similar data were reported by the Cystic Fibrosis Foundation,^1^ which has 28,983 CF individuals enrolled, being 49.3% up to 18 years and showing that mean BMI/age percentiles in children with CF increased from 40.3 in 2010 to 54.2 in 2015. In 2015, among patients treated, 0.5% developed HS and 2.3% had cirrhosis up to 18 years. These data were obtained by survey and from the literature on the frequency of HS in CF patients.

The Brazilian Registry of Cystic Fibrosis (REBRAFC),^23^ coordinated by the Brazilian Group of Cystic Fibrosis Studies (GBEFC), holds demographic data related to the diagnosis and treatment of these patients. With 3,511 patients registered by 2014, 75% are under 18 years, with average of 11.5 years. Regarding nutritional status, data show that patients’ nutritional status is inadequate, with a mean BMI percentile of 21.3 (BMI below the 50th percentile). Among patients evaluated, 8.8% presented some degree of hepatic involvement, 1% had cirrhosis, and 0.04% required liver transplantation in 2014.

A study conducted in Rio Grande do Sul with 82 patients aged 7 months to 16 years showed that 26.8% of the sample was malnourished by BMI <10 percentile as cutoff for malnutrition.^24^ Another study with 85 patients, with mean of age 11.2±3.2, reported a 22.3% prevalence of malnourished patients considering BMI below the 25th percentile as cutoff point.^25^

Nutritional status in our study was assessed according to WHO standards. Other studies have used the recommendations by the international consensuses of CF, comparing divergent studies, since there is a reduction in the number of individuals considered eutrophic and an increase in cases of malnutrition. This was identified in another study.^26^ There are reports of research relating liver disease and nutritional status, but taking into account several aspects of liver involvement, from elevation of liver enzymes to cirrhosis. In these studies, no association between liver disease and nutritional status was found.^27^ ^,^ ^28^ As an exception, there is one recent study by Ayoub et al.^29^ that analyzed the risk factors for HS in adult CF patients, but results could not be compared because patients were within a wide range of age (median age 29 years), and also because the authors found association between overweight and HS and concluded that HS in adult CF patients shares similarities with nonalcoholic fatty liver disease.

Malnutrition is multifactorial in CF, including poor dietary intake, increased daily energy requirement and is associated with poor nutrient digestion. Patients diagnosed in newborn screening programs benefit from early intervention, which is associated with positive nutritional status.^30^ Early intervention in CF can maintain the patient’s good nutritional status and minimize the effects of the malnutrition-infection vicious cycle. Keeping track of secondary HS complications are a must, as these are associated with increased morbidity and mortality, which directly affect patient’s health and quality of life.

One of the limitations of this study was not evaluating body composition by electrical bioimpedance, to better interpret anthropometric measures, which showed greater reserve of lean mass in HS group. Another limitation was the lack of a survey on eating habits to identify unbalanced diet and use of dietary supplements that could interfere with nutritional status. Another limitation was the small sample, composed by subjects selected by convenience sampling.

In conclusion, the frequency of HS is high in patients with CF and it is not associated with malnutrition, according to the parameters of BMI, TSF and MAMC. MAC and MAMC values indicated greater reserve of muscle mass in patients with HS.
